# A Verified SAT Solver Framework with Learn, Forget, Restart, and Incrementality

**DOI:** 10.1007/s10817-018-9455-7

**Published:** 2018-03-12

**Authors:** Jasmin Christian Blanchette, Mathias Fleury, Peter Lammich, Christoph Weidenbach

**Affiliations:** 1 Section of Theoretical Computer Science, Department of Computer Science, Vrije Universiteit Amsterdam, De Boelelaan 1081a, 1081 HV Amsterdam, The Netherlands; 2 Max-Planck-Institut für Informatik, Saarland Informatics Campus E1 4, 66123 Saarbrücken, Germany; 3 Saarbrücken Graduate School of Computer Science, Saarland Informatics Campus E1 3, 66123 Saarbrücken, Germany; 4 Institut für Informatik, Technische Universität München, Boltzmannstraße 3, Garching, Germany

**Keywords:** SAT solvers, CDCL, DPLL, Proof assistants, Isabelle/HOL

## Abstract

We developed a formal framework for conflict-driven clause learning (CDCL) using the Isabelle/HOL proof assistant. Through a chain of refinements, an abstract CDCL calculus is connected first to a more concrete calculus, then to a SAT solver expressed in a functional programming language, and finally to a SAT solver in an imperative language, with total correctness guarantees. The framework offers a convenient way to prove metatheorems and experiment with variants, including the Davis–Putnam–Logemann–Loveland (DPLL) calculus. The imperative program relies on the two-watched-literal data structure and other optimizations found in modern solvers. We used Isabelle’s Refinement Framework to automate the most tedious refinement steps. The most noteworthy aspects of our work are the inclusion of rules for forget, restart, and incremental solving and the application of stepwise refinement.

## Introduction

Researchers in automated reasoning spend a substantial portion of their work time developing logical calculi and proving metatheorems about them. These proofs are typically carried out with pen and paper, which is error-prone and can be tedious. Today’s proof assistants are easier to use than their predecessors and can help reduce the amount of tedious work, so it makes sense to use them for this kind of research.

In this spirit, we started an effort, called  (Isabelle Formalization of Logic) [4], that aims at developing libraries and a methodology for formalizing modern research in the field, using the Isabelle/HOL proof assistant [45, 46]. Our initial emphasis is on established results about propositional and first-order logic. In particular, we are formalizing large parts of Weidenbach’s forthcoming textbook, tentatively called  . Our inspiration for formalizing logic is the  (Isabelle Formalization of Rewriting) project [55], which focuses on term rewriting.

The objective of formalization work is not to eliminate paper proofs, but to complement them with rich formal companions. Formalizations help catch mistakes, whether superficial or deep, in specifications and theorems; they make it easy to experiment with changes or variants of concepts; and they help clarify concepts left vague on paper.

This article presents our formalization of CDCL (conflict-driven clause learning) based on  , derived as a refinement of Nieuwenhuis, Oliveras, and Tinelli’s abstract presentation of CDCL [43]. It is the algorithm implemented in modern propositional satisfiability (SAT) solvers. We start with a family of formalized abstract DPLL (Davis–Putnam–Logemann–Loveland) [17] and CDCL [3, 6, 40, 42] transition systems from Nieuwenhuis et al. (Sect. 3). Some of the calculi include rules for learning and forgetting clauses and for restarting the search. All calculi are proved sound and complete, as well as terminating under a reasonable strategy.

The abstract CDCL calculus is refined into the more concrete calculus presented in  and recently published [57] (Sect. 4). The latter specifies a criterion for learning clauses representing first unique implication points [6, Chapter 3], with the guarantee that learned clauses are not redundant and hence derived at most once. The correctness results (soundness, completeness, termination) are inherited from the abstract calculus. The calculus also supports incremental solving.

The concrete calculus is refined further to obtain a verified, but very naive, functional program extracted using Isabelle’s code generator (Sect. 5). The final refinement step derives an imperative SAT solver implementation with efficient data structures, including the well-known two-watched-literal optimization (Sect. 6).

Our work is related to other verifications of SAT solvers, which largely aimed at increasing their trustworthiness (Sect. 7). This goal has lost some of its significance with the emergence of formats for certificates that are easy to generate, even in highly optimized solvers, and that can be processed efficiently by verified checkers [16, 33]. In contrast, our focus is on formalizing the metatheory of CDCL, with the following objectives:

- Develop a basic library of formalized results and a methodology aimed at researchers who want to experiment with calculi.
- Study and connect the members of the CDCL family, including newer extensions.
- Check the proofs in  and provide a formal companion to the book.
- Assess the suitability of Isabelle/HOL for formalizing logical calculi.

Compared with the other verified SAT solvers, the most noteworthy features of our framework are the inclusion of rules for forget, restart, and incremental solving and the application of stepwise refinement [59] to transfer results. The framework is available as part of the  repository [20].

Any formalization effort is a case study in the use of a proof assistant. We depended heavily on the following features of Isabelle:

- *Isar* [58] is a textual proof format inspired by the pioneering Mizar system [41]. It makes it possible to write structured, readable proofs—a requisite for any formalization that aims at clarifying an informal proof.
- *Sledgehammer* [7, 48] integrates superposition provers and SMT (satisfiability modulo theories) solvers in Isabelle to discharge proof obligations. The SMT solvers, and one of the superposition provers [56], are built around a SAT solver, resulting in a situation where SAT solvers are employed to prove their own metatheory.
- *Locales* [2, 25] parameterize theories over operations and assumptions, encouraging a modular style. They are useful to express hierarchies of concepts and to reduce the number of parameters and assumptions that must be threaded through a formal development.
- The *Refinement Framework* [30] can be used to express refinements from abstract data structures and algorithms to concrete, optimized implementations. This allows us to reason about simple algebraic objects and yet obtain efficient programs. The *Sepref* tool [31] builds on the Refinement Framework to derive an imperative program, which can be extracted to Standard ML and other programming languages. For example, Isabelle’s algebraic lists can be refined to mutable arrays in ML.

An earlier version of this work was presented at IJCAR 2016 [11]. This article extends the conference paper with a description of the refinement to an imperative implementation (Sects. 2.4 and 6) and of the formalization of Weidenbach’s DPLL calculus (Sect. 4.1). To make the paper more accessible, we expanded the background material about Sledgehammer (Sect. 2.1) and Isar (Sect. 2.2).

## Isabelle/HOL

Isabelle [45, 46] is a generic proof assistant that supports several object logics. The metalogic is an intuitionistic fragment of higher-order logic (HOL) [15]. The types are built from type variables  and *n*-ary type constructors, normally written in postfix notation (e.g, ). The infix type constructor  is interpreted as the (total) function space from  to . Function applications are written in a curried style without parentheses (e.g., ). Anonymous functions $x \mapsto t_x$ are written $\lambda x.\; t_x$. The notation  indicates that term *t* has type $\tau$. Propositions are terms of type , a type with at least two values. Symbols belonging to the signature (e.g., ) are uniformly called *constants*, even if they are functions or predicates. No syntactic distinction is enforced between terms and formulas. The metalogical operators are universal quantification , implication , and equality . The notation ${\bigwedge }x.\; p_x$ abbreviates ${\bigwedge }\;(\lambda x.\; p_x)$ and similarly for other binder notations.

Isabelle/HOL is the instantiation of Isabelle with HOL, an object logic for classical HOL extended with rank-1 (top-level) polymorphism and Haskell-style type classes. It axiomatizes a type  of Booleans as well as its own set of logical symbols ($\forall$, $\exists$,  ,  , $\lnot$, $\wedge$, $\vee$, $\longrightarrow$, , $=$). The object logic is embedded in the metalogic via a constant , which is normally not printed. In practice, the distinction between the two logical levels is important operationally but not semantically.

Isabelle adheres to the tradition that started in the 1970s by the LCF system [22]: All inferences are derived by a small trusted kernel; types and functions are defined rather than axiomatized to guard against inconsistencies. High-level specification mechanisms let us define important classes of types and functions, notably inductive datatypes, inductive predicates, and recursive functions. Internally, the system synthesizes appropriate low-level definitions and derives the user specifications via primitive inferences.

Isabelle developments are organized as collections of theory files that build on one another. Each file consists of definitions, lemmas, and proofs expressed in Isar [58], Isabelle’s input language. Isar proofs are expressed either as a sequence of tactics that manipulate the proof state directly or in a declarative, natural-deduction format inspired by Mizar. Our formalization almost exclusively employs the more readable declarative style.

### Sledgehammer

The Sledgehammer subsystem [7, 48] integrates automatic theorem provers in Isabelle/HOL, including CVC4, E, LEO-II, Satallax, SPASS, Vampire, veriT, and Z3. Upon invocation, it heuristically selects relevant lemmas from the thousands available in loaded libraries, translates them along with the current proof obligation to SMT-LIB or TPTP, and invokes the automatic provers. In case of success, the machine-generated proof is translated to an Isar proof that can be inserted into the formal development, so that the external provers do not need to be trusted.

Sledgehammer is part of most Isabelle users’ workflow, and we invoke it dozens of times a day (according to the log files it produces). For example, while formalizing some results that depend on multisets, we found ourselves needing the basic property 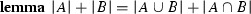 where *A* and *B* are finite multisets, $\cup$ denotes union defined such that for each element *x*, the multiplicity of *x* in $A \cup B$ is the maximum of the multiplicities of *x* in *A* and *B*, $\cap$ denotes intersection, and  denotes cardinality. This lemma was not available in Isabelle’s underdeveloped multiset library, so we invoked Sledgehammer. Within 30 s, the tool came back with a brief proof text invoking a suitable tactic with a list of ten lemmas from the library, which we could insert into our formalization: 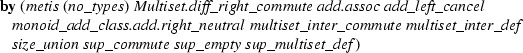 The generated proof refers to 10 library lemmas by name and applies the *metis* search tactic.

### Isar

Without Sledgehammer, proving the above property could easily have taken 5–15 min. A manual proof, expressed in Isar’s declarative style, might look like this: 
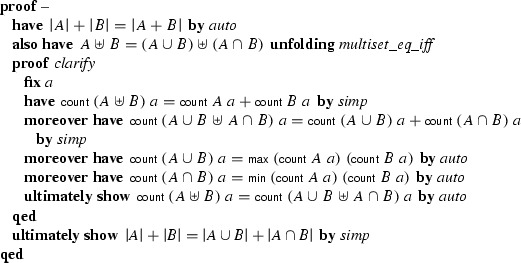
 The  function returns the multiplicity of an element in a multiset. The $\uplus$ operator denotes the disjoint union operation—for each element, it computes the sum of the multiplicities in the operands (as opposed to the maximum for $\cup$).

In Isar proofs, intermediate properties are introduced using  and proved using a tactic such as *simp* and *auto*. Proof blocks (  $\;\ldots \;$  ) can be nested. The advantage of Isar proofs over one-line *metis* proofs is that we can follow and understand the steps. However, for lemmas about multisets and other background theories, we are usually content if we can get a proof automatic and carry on with formalizing the more interesting foreground theory.

### Locales

Isabelle locales are a convenient mechanism for structuring large proofs. A locale fixes types, constants, and assumptions within a specified scope. A schematic example follows: 
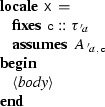
 The definition of locale  implicitly fixes a type , explicitly fixes a constant  whose type  may depend on , and states an assumption  over  and . Definitions made within the locale may depend on  and , and lemmas proved within the locale may additionally depend on . A single locale can introduce several types, constants, and assumptions. Seen from the outside, the lemmas proved in  are polymorphic in type variable , universally quantified over , and conditional on .

Locales support inheritance, union, and embedding. To embed  into  , or make  a *sublocale* of  , we must recast an instance of  into an instance of  , by providing, in the context of  , definitions of the types and constants of  together with proofs of  ’s assumptions. The command 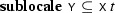 emits the proof obligation , where $\upsilon$ and  may depend on types and constants available in  . After the proof, all the lemmas proved in  become available in  , with  and  instantiated with $\upsilon$ and .

### Refinement Framework

The Refinement Framework [30] provides definitions, lemmas, and tools that assist in the verification of functional and imperative programs via stepwise refinement [59]. The framework defines a programming language that is built on top of a nondeterminism monad. A program is a function that returns an object of type : 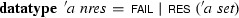 The Isabelle syntax is similar to that of Standard ML and other typed functional programming languages: The type is freely generated by its two constructors,  and . The set *X* in  specifies the possible values that can be returned. The return statement is defined as a constant  and specifies a single value, whereas  indicates that an unspecified positive number is returned. The simplest program is a semantic specification of the possible outputs, encapsulated in a  constructor. The following example is a nonexecutable specification of the function that subtracts 1 from every element of the list  (with $0 - 1$ defined as 0 on natural numbers): 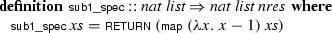 Program refinement uses the same source and target language. The refinement relation $\le$ is defined by  and  for all *r*. For example, the concrete program  refines ($\le$) the abstract program , meaning that all concrete behaviors are possible in the abstract version. The bottom element  is an unrefinable program; the top element  represents a run-time failure (e.g., a failed assertion) or divergence.

Refinement can be used to change the program’s data structures and algorithms, towards a more deterministic and usually more efficient program for which executable code can be generated. For example, we can refine the previous specification to a program that uses a ‘while’ loop: 
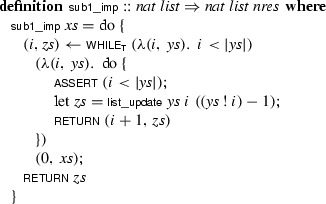
 The program relies on the following constructs:

- The ‘do’ construct is a convenient Haskell-inspired syntax for expressing monadic computations (here, on the nondeterminism monad).
- The  combinator takes a condition, a loop body, and a start value. In our example, the loop’s state is a pair of the form . The  subscript in the combinator’s name indicates that the loop must not diverge. Totality is necessary for code generation.
- The  statement takes an assertion that must always be true when the statement is executed.
- The  operation returns the $(i + 1)$st element of , and  replaces the $(i + 1)$st element by *y*.

To prove the refinement lemma , we can use the *refine_vcg* proof method provided by the Refinement Framework. This method heuristically aligns the statements of the two programs and generates proof obligations, which are passed to the user. If the abstract program has the form  or , as is the case here, *refine_vcg* applies Hoare-logic-style rules to generate the verification conditions. For our example, two of the resulting proof obligations correspond to the termination of the ‘while’ loop and the correctness of the assertion. We can use the measure  to prove termination.

In a refinement step, we can also change the types. For our small program, if we assume that the natural numbers in the list are all nonzero, we can replace them by integers and use the subtraction operation on integers (for which $0 - 1 = -1 \not = 0$). The program remains syntactically identical except for the type annotation: 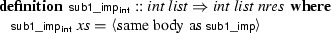


We want to establish the following relation: If all elements in  are nonzero and the elements of  are positionwise numerically equal to those of , then any list of integers returned by  is positionwise numerically equal to some list returned by . The framework lets us express preconditions and connections between types using higher-order relations called relators: 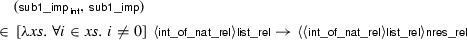
The relation  relates natural numbers with their integer counterparts (e.g., ). The syntax of relators mimics that of types; for example, if *R* is the relation for , then  is the relation for , and  is the relation for . The ternary relator $[p]\,R \rightarrow S$, for functions , lifts the relations *R* and *S* for  and  under precondition *p*.

The *Imperative HOL* library [14] defines a heap monad that can express imperative programs with side effects. On top of Imperative HOL, a separation logic, with assertion type , can be used to express relations  between plain values, of type , and data structures on the heap, of type . For example,  relates lists of  elements with mutable arrays of  elements, where  is used to relate the elements. The relation between the ! operator on lists and its heap-based counterpart  can be expressed as follows: 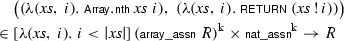 The arguments’ relations are annotated with $^{\mathrm {k}}$ (“keep”) or $^{\mathrm {d}}$ (“destroy”) superscripts that indicate whether the previous value can still be accessed after the operation has been performed. Reading an array leaves it unchanged, whereas updating it destroys the old array.

The *Sepref* tool automates the transition from the nondeterminism monad to the heap monad. It keeps track of the values that are destroyed and ensures that they are not used later in the program. Given a suitable source program, it can automatically generate the target program and prove the corresponding refinement lemma automatically. The main difficulty is that some low-level operations have side conditions, which we must explicitly discharge by adding assertions at the right points in the source program to guide Sepref.

The following command generates a heap program called  from the source program : 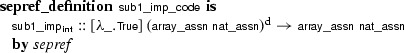 The generated array-based program is 
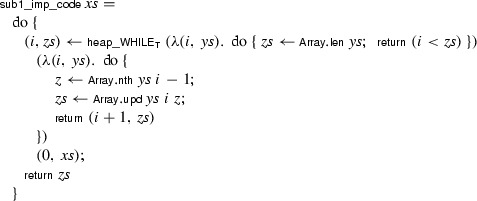
 The end-to-end refinement theorem, obtained by composing the refinement lemmas, is 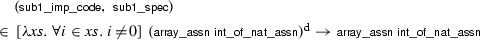


If we want to execute the program efficiently, we can translate it to Standard ML using Isabelle’s code generator [23]. The following imperative code, including its dependencies, is generated (in slightly altered form):

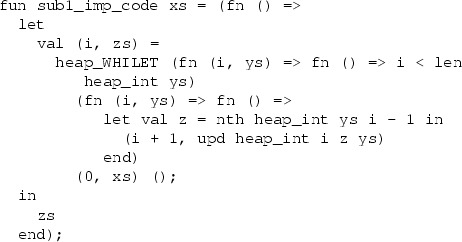

The ML idiom $\texttt {(fn () => \ldots ) ()}$ is inserted to delay the evaluation of the body, so that the side effects occur in the intended order.

## Abstract CDCL

The abstract CDCL calculus by Nieuwenhuis et al. [43] forms the first layer of our refinement chain. The formalization relies on basic Isabelle libraries for lists and multisets and on custom libraries for propositional logic. Properties such as partial correctness and termination (given a suitable strategy) are inherited by subsequent layers.

### Propositional Logic

The DPLL and CDCL calculi distinguish between literals whose truth value has been decided arbitrarily and those that are entailed by the current decisions; for the latter, it is sometimes useful to know which clause entails it. To capture this information, we introduce a type of annotated literals, parameterized by a type  of propositional variables and a type  of clauses: 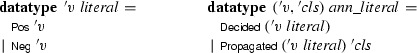


The simpler calculi do not use  ; they take , a singleton type whose unique value is (). Informally, we write *A*, $\lnot \,A$, and $L^\dag$ for positive, negative, and decision literals, and we write $L^C$ (with ) or simply *L* (if  or if the clause *C* is irrelevant) for propagated literals. The unary minus operator is used to negate a literal, with $- (\lnot \,A) = A$.

As is customary in the literature [1, 57], clauses are represented by multisets, ignoring the order of literals but not repetitions. A  is a (finite) multiset over . Clauses are often stored in sets or multisets of clauses. To ease reading, we write clauses using logical symbols (e.g., $\bot$, *L*, and $C \vee D$ for $\emptyset$, $\{L\}$, and $C \uplus D$). Given a clause *C*, we write $\lnot \,C$ for the formula that corresponds to the clause’s negation.

Given a set or multiset *I* of literals, $I \vDash C$ is true if and only if *C* and *I* share a literal. This is lifted to sets and multisets of clauses or formulas: . A set or multiset is satisfiable if there exists a consistent set or multiset of literals *I* such that $I \vDash N$. Finally,  These notations are also extended to formulas.

### DPLL with Backjumping

Nieuwenhuis et al. present CDCL as a set of transition rules on states. A state is a pair , where *M* is the *trail* and *N* is the multiset of clauses to satisfy. In a slight abuse of terminology, we will refer to the multiset of clauses as the “clause set.” The trail is a list of annotated literals that represents the partial model under construction. The empty list is written . Somewhat nonstandardly, but in accordance with Isabelle conventions for lists, the trail grows on the left: Adding a literal *L* to *M* results in the new trail $L \cdot M$, where . The concatenation of two lists is written $M \mathbin {@} M'$. To lighten the notation, we often build lists from elements and other lists by simple juxtaposition, writing $M L M'$ for $M \mathbin {@} L \cdot M'$.

The core of the CDCL calculus is defined as a transition relation , an extension of classical DPLL [17] with nonchronological backtracking, or *backjumping*. The  part of the name refers to Nieuwenhuis, Oliveras, and Tinelli. The calculus consists of three rules, starting from an initial state . In the following, we abuse notation, implicitly converting $\vDash$’s first operand from a list to a set and ignoring annotations on literals:

  if *N* contains a clause $C\vee L$ such that $M \vDash \lnot \, C$ and *L* is undefined in *M* (i.e., neither $M \vDash L$ nor $M \vDash - L$)
  if the atom of *L* occurs in *N* and is undefined in *M*
  if *N* contains a conflicting clause *C* (i.e., $M'L^\dag M\vDash \lnot \, C$) and there exists a clause $C'\vee L'$ such that $N\vDash C'\vee L'$, $M \vDash \lnot \, C'$, and $L'$ is undefined in *M* but occurs in *N* or in $M'L^\dag$

The  rule is more general than necessary for capturing DPLL, where it suffices to negate the leftmost decision literal. The general rule can also express nonchronological backjumping, if $C'\vee L'$ is a new clause derived from *N* (but not necessarily in *N*).

We represented the calculus as an inductive predicate. For the sake of modularity, we formalized the rules individually as their own predicates and combined them to obtain : 
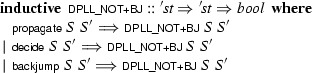
 Since there is no call to  in the assumptions, we could also have used a plain  here, but the  command provides convenient introduction and elimination rules. The predicate operates on states of type . To allow for refinements, this type is kept as a parameter of the calculus, using a locale that abstracts over it and that provides basic operations to manipulate states: 
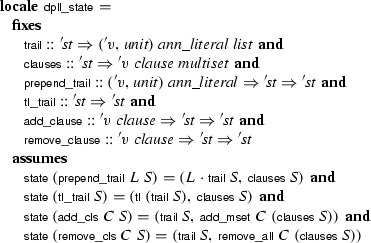
 where  converts an abstract state of type  to a pair (*M*, *N*). Inside the locale, states are compared extensionally:  is true if the two states have identical trails and clause sets (i.e., if ). This comparison ignores any other fields that may be present in concrete instantiations of the abstract state type .

Each calculus rule is defined in its own locale, based on  and parameterized by additional side conditions. Complex calculi are built by inheriting and instantiating locales providing the desired rules. For example, the following locale provides the predicate corresponding to the  rule, phrased in terms of an abstract DPLL state: 
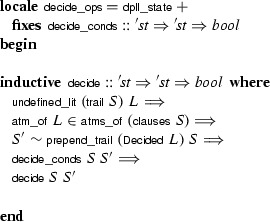


Following a common idiom, the  calculus is distributed over two locales: The first locale,   , defines the  calculus; the second locale,  , extends it with an assumption expressing a structural invariant over  that is instantiated when proving concrete properties later. This cannot be achieved with a single locale, because definitions may not precede assumptions.

#### Theorem 1

(Termination [20,  ]) The relation  is well founded.

Termination is proved by exhibiting a well-founded relation $\prec$ such that  whenever . Let  and  with the decompositions

$$\begin{aligned} M&= \smash {M_n L_n^\dag \cdots M_1 L_1^\dag M_0}&M'&= \smash {M'_{n'} \smash {L'}_{\!\!n\smash {'}}^\dag \cdots M'_1 \smash {L'}_{\!\!\!1}^\dag M'_0} \end{aligned}$$

where the trail segments $M_0,\ldots ,M_n,M'_0,\ldots ,M'_{n\smash {'}}$ contain no decision literals. Let *V* be the number of distinct variables occurring in the initial clause set *N*. Now, let $\nu \,M = V - \left| M\right|$, indicating the number of unassigned variables in the trail *M*. Nieuwenhuis et al. define $\prec$ such that  if

1. there exists an index $i \le n, n'$ such that $[\nu \, M'_0,\, \cdots ,\, \nu \, M'_{i-1}] = [\nu \, M_0,\, \cdots ,\, \nu \, M_{i-1}]$ and $\nu \,M'_i < \nu \,M_i$; or
2. $[\nu \, M_0,\, \cdots ,\, \nu \, M_{n}]$ is a strict prefix of $[\nu \, M'_0,\, \cdots ,\, \nu \, M'_{n'}]$.

This order is not to be confused with the lexicographic order: We have  by condition (2), whereas . Yet the authors justify well-foundedness by appealing to the well-foundedness of  on bounded lists over finite alphabets. In our proof, we clarify and simplify matters by mapping states  to lists $\bigl [\left| M_0\right| , \cdots ,\left| M_n\right| \bigr ]$, without appealing to $\nu$. Using the standard lexicographic order, states become *larger* with each transition: 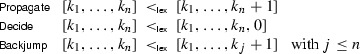 The lists corresponding to possible states are bounded by the list $[V, \dots , V]$ consisting of *V* occurrences of *V*,  thereby delimiting a finite domain $D = \{[k_1,\ldots ,k_n] \mid k_1,\cdots ,k_n,n \le V\}$. We take $\prec$ to be the restriction of  to *D*. A variant of this approach is to encode lists into a measure  and let , building on the well-foundedness of > over bounded sets of natural numbers.

A *final* state is a state from which no transitions are possible. Given a relation , we write  for the right-restriction of its reflexive transitive closure to final states (i.e.,  if and only if ).

#### Theorem 2

(Partial Correctness [20,  ]) If , then *N* is satisfiable if and only if $M\vDash N.$

We first prove structural invariants on arbitrary states  reachable from , namely: (1) each variable occurs at most once in $M'$; (2) if $M' = M_2 L M_1$ where *L* is propagated, then $M_1, N \vDash L$. From these invariants, together with the constraint that  is a final state, it is easy to prove the theorem.

### Classical DPLL

The locale machinery allows us to derive a classical DPLL calculus from DPLL with backjumping. We call this calculus . It is achieved through a  locale that restricts the Backjump rule so that it performs only chronological backtracking:

  if *N* contains a conflicting clause and $M'$ contains no decision literals

Because of the locale parameters,  is strictly speaking a family of calculi.

#### Lemma 3

(Backtracking [20,  ]) The  rule is a special case of the  rule.

The  rule depends on two clauses: a conflict clause *C* and a clause $C'\vee L'$ that justifies the propagation of $L'\!.$ The conflict clause is specified by  . As for $C'\vee L'$, given a trail $M'L^\dag M$ decomposable as $M_nL^\dag M_{n-1}L_{n\smash {-1}}^\dag \cdots M_1 L_1^ \dag M_0$ where $M_0,\cdots ,M_n$ contain no decision literals, we can take $C' = -L_1\vee \cdots \vee -L_{n-1}$.

Consequently, the inclusion  holds. In Isabelle, this is expressed as a locale instantiation:  is made a sublocale of , with a side condition restricting the application of the  rule. The partial correctness and termination theorems are inherited from the base locale.  instantiates the abstract state type  with a concrete type of pairs. By discharging the locale assumptions emerging with the  command, we also verify that these assumptions are consistent. Roughly: 
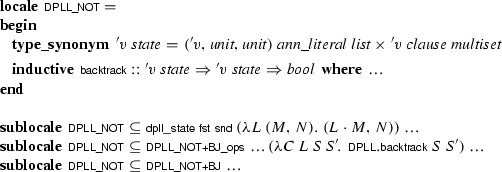


If a conflict cannot be resolved by backtracking, we would like to have the option of stopping even if some variables are undefined. A state  is *conclusive* if $M \vDash N$ or if *N* contains a conflicting clause and *M* contains no decision literals. For , all final states are conclusive, but not all conclusive states are final.

#### Theorem 4

(Partial Correctness [20,  ])

If   and  is a conclusive state, *N* is satisfiable if and only if $M\vDash N$.

The theorem does not require stopping at the first conclusive state. In an implementation, testing $M\vDash N$ can be expensive, so a solver might fail to notice that a state is conclusive and continue for some time. In the worst case, it will stop in a final state—which is guaranteed to exist by Theorem 1. In practice, instead of testing whether $M\vDash N$, implementations typically apply the rules until every literal is set. When *N* is satisfiable, this produces a total model.

### The CDCL Calculus

The abstract CDCL calculus extends  with a pair of rules for learning new lemmas and forgetting old ones:

   if $N\vDash C$ and each atom of *C* is in *N* or *M*
   if $$N\vDash C$$   

In practice, the  rule is normally applied to clauses built exclusively from atoms in *M*, because the learned clause is false in *M*. This property eventually guarantees that the learned clause is not redundant (e.g., it is not already contained in *N*).

We call this calculus . In general,  does not terminate, because it is possible to learn and forget the same clause infinitely often. But for some instantiations of the parameters with suitable restrictions on  and  , the calculus always terminates.

#### Theorem 5

(Termination [20,  ])

   Let  be an instance of the  calculus (i.e., ). If  admits no infinite chains consisting exclusively of  and  transitions, then  is well founded.

In many SAT solvers, the only clauses that are ever learned are the ones used for backtracking. If we restrict the learning so that it is always done immediately before backjumping, we can be sure that some progress will be made between a  and the next  or  . This idea is captured by the following combined rule:

  if , *L*, , *M*, , *N* satisfy  ’s side conditions

The calculus variant that performs this rule instead of  and  is called  . Because a single  transition corresponds to two transitions in , the inclusion  does not hold. Instead, we have . Each step of  corresponds to a single step in  or a two-step sequence consisting of  followed by  .

### Restarts

Modern SAT solvers rely on a dynamic decision literal heuristic. They periodically restart the proof search to apply the effects of a changed heuristic. This helps the calculus focus on a part of the initial clauses where it can make progress. Upon a restart, some learned clauses may be removed, and the trail is reset to . Since our calculus has a  rule, the  rule needs only to clear the trail. Adding  to  yields  . However, this calculus does not terminate, because  can be applied infinitely often.

**Fig. 1:**
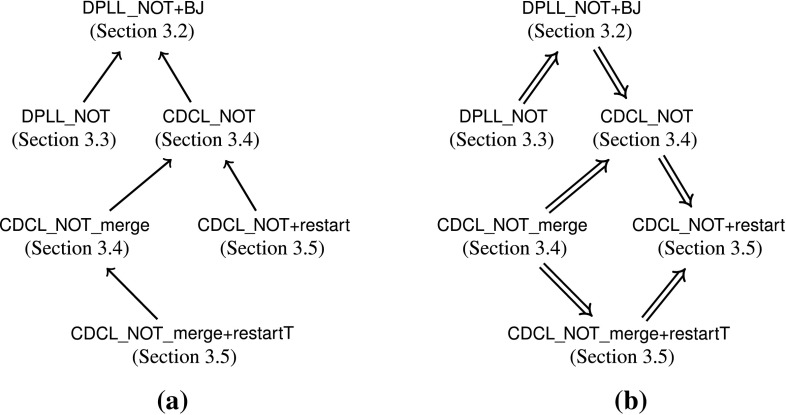
Connections between the abstract calculi. **a** Syntactic dependencies. **b** Refinements

A working strategy is to gradually increase the number of transitions between successive restarts. This is formalized via a locale parameterized by a base calculus  and an unbounded function . Nieuwenhuis et al. require *f* to be strictly increasing, but unboundedness is sufficient.

The extended calculus  operates on states of the form , where  is a state in the base calculus and *n* counts the number of restarts. To simplify the presentation, we assume that bases states  are pairs (*M*, *N*). The calculus  starts in the state  and consists of two rules:

   if  and $$m \ge f\>n$$
   if

The symbol  represents the base calculus  transition relation, and  denotes an *m*-step transition in  . The  in  reminds us that we count the number of *transitions*; in Sect. 4.5, we will review an alternative strategy based on the number of conflicts or learned clauses. Termination relies on a measure $\mu _V$ associated with  that may not increase from restart to restart: If , then . The measure may depend on *V*,  the number of variables occurring in the problem.

We instantiated the locale parameter  with  and *f* with the Luby sequence ($1, 1, 2, 1, 1, 2, 4, \cdots$) [35], with the restriction that no clause containing duplicate literals is ever learned, thereby bounding the number of learnable clauses and hence the number of transitions taken by  .

Figure 1a summarizes the syntactic dependencies between the calculi reviewed in this section. An arrow  indicates that  is defined in terms of . Figure 1b presents the refinements between the calculi. An arrow  indicates that we proved  or some stronger result—either by locale embedding (  ) or by simulating  ’s behavior in terms of  .

## A Refined CDCL Towards an Implementation

The  calculus captures the essence of modern SAT solvers without imposing a policy on when to apply specific rules. In particular, the  rule depends on a clause $C' \vee L'$ to justify the propagation of a literal, but does not specify a procedure for coming up with this clause. For  , Weidenbach developed a calculus that is more specific in this respect, and closer to existing solver implementations, while keeping many aspects unspecified [57]. This calculus,  , is also formalized in Isabelle and connected to  .

### The New DPLL Calculus

Independently from the previous section, we formalized DPLL as described in  . The calculus operates on states (*M*, *N*), where *M* is the trail and *N* is the initial clause set. It consists of three rules:

   if $C\vee L \in N \uplus U$, $M \vDash \lnot \, C$, and *L* is undefined in *M*
   if *L* is undefined in *M* and occurs in *N*
  if *N* contains a conflicting clause and $M'$ contains no decision literals

performs chronological backtracking: It undoes the last decision and picks the opposite choice. Conclusive states for  are defined as for  (Sect. 3.3).

The termination and partial correctness proofs given by Weidenbach depart from Nieuwenhuis et al. We also formalized them:

#### Theorem 6

(Termination [20,  ]) The relation  is well founded.

Termination is proved by exhibiting a well-founded relation that includes . Let *V* be the number of distinct variables occurring in the clause set *N*. The weight $\nu \,L$ of a literal *L* is 2 if *L* is a decision literal and 1 otherwise. The measure isLists are compared using the lexicographic order, which is well founded because there are finitely many literals and all lists have the same length. It is easy to check that the measure decreases with each transition:
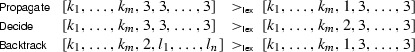


#### Theorem 7

(Partial Correctness [20,  ]) If  and  is a conclusive state, *N* is satisfiable if and only if $M\vDash N.$

The proof is analogous to the proof of Theorem 2. Some lemmas are shared between both proofs. Moreover, we can link Weidenbach’s DPLL calculus with the version we derived from  in Sect. 3.3:

#### Theorem 8

(DPLL [20,  ]) For all states  that satisfy basic structural invariants,  if and only if 

This provides another way to establish Theorems 6 and 7. Conversely, the simple measure that appears in the above termination proof can also be used to establish the termination of the more general  calculus (Theorem 1).

### The New CDCL Calculus

The  calculus operates on states , where *M* is the trail; *N* and *U* are the sets of initial and learned clauses, respectively; and *D* is a conflict clause, or the distinguished clause $\top$ if no conflict has been detected.

In the trail *M*,  each decision literal *L* is marked as such ($L^\dag$—i.e., ), and each propagated literal *L* is annotated with the clause *C* that caused its propagation ($L^C$—i.e., ). The level of a literal *L* in *M* is the number of decision literals to the right of the atom of *L* in *M*, or 0 if the atom is undefined. The level of a clause is the highest level of any of its literals, with 0 for $\bot$, and the level of a state is the maximum level (i.e., the number of decision literals). The calculus assumes that *N* contains no clauses with duplicate literals and never produces clauses containing duplicates.

The calculus starts in a state . The following rules apply as long as no conflict has been detected:

  if $C\vee L \in N \uplus U$, $M \vDash \lnot \, C$, and *L* is undefined in *M*
   if *L* is undefined in *M* and occurs in *N*
   if $$D \in N \uplus U$$ and $$M \vDash \lnot \, D$$
   if $$M \not \vDash N$$
   if $M \not \vDash N$ and *M* contains no literal $L^C$

The  and  rules generalize their  counterparts. Once a conflict clause has been detected and stored in the state, the following rules cooperate to reduce it and backtrack, exploring a first unique implication point [6, Chapter 3]:

   if $D \notin \{\bot ,\top \}$ and $-L$ does not occur in *D*
  if *D* has the same level as the current state
  if *L* has the level of the current state, *D* has a lower level, and *K* and *D* have the same level

Exhaustive application of these three rule corresponds to a single step by the combined learning and nonchronological backjumping rule  from  . The  rule is even more general and can be used to express learned clause minimization [54].

In  , $C \cup D$ is the same as $C \vee D$ (i.e., $C \uplus D$), except that it keeps only one copy of the literals that belong to both *C* and *D*. When performing propagations and processing conflict clauses, the calculus relies on the invariant that clauses never contain duplicate literals. Several other structural invariants hold on all states reachable from an initial state, including the following: The clause annotating a propagated literal of the trail is a member of $N \uplus U.$ Some of the invariants were not mentioned in the textbook (e.g., whenever $L^C$ occurs in the trail, *L* is a literal of *C*). Formalization helped develop a better understanding of the data structure and clarify the book.

Like  ,  has a notion of conclusive state. A state  is *conclusive* if $D = \top$ and $M\vDash N$ or if $D = \bot$ and *N* is unsatisfiable. The calculus always terminates, but without a suitable strategy, it can block in an inconclusive state. At the end of the following derivation, neither  nor  can process the conflict further: 
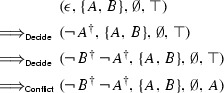


### A Reasonable Strategy

To prove correctness, we assume a *reasonable* strategy:  and  are preferred over  ;  and  are not applied. (We will lift the restriction on  and  in Sect. 4.5.) The resulting calculus, , refines  with the assumption that derivations are produced by a reasonable strategy. This assumption is enough to ensure that the calculus can backjump after detecting a nontrivial conflict clause other than $\bot$. The crucial invariant is the existence of a literal with the highest level in any conflict, so that  can be applied. The textbook suggests preferring  to  and  to the other rules. While this makes sense in an implementation, it is not needed for any of our metatheoretical results.

#### Theorem 9

(Partial Correctness [20,  ]) If   and *N* contains no clauses with duplicate literals,  is a conclusive state.

Once a conflict clause has been stored in the state, the clause is first reduced by a chain of  and  transitions. Then, there are two scenarios: (1) the conflict is solved by a  , at which point the calculus may resume propagating and deciding literals; (2) the reduced conflict is $\bot$, meaning that *N* is unsatisfiable—i.e., for unsatisfiable clause sets, the calculus generates a resolution refutation.

The  calculus is designed to have respectable complexity bounds. One of the reasons for this is that the same clause cannot be learned twice:

#### Theorem 10

(No Relearning [20,  ]) 

  If we have   then no  transition is possible from the latter state causing the addition of a clause from $N \uplus U$ to *U*.

The formalization of this theorem posed some challenges. The informal proof in  is as follows (with slightly adapted notations):

*Proof * By contradiction. Assume CDCL learns the same clause twice, i.e., it reaches a state  where  is applicable and  More precisely, the state has the form  where the $K_i$, $i>1$ are propagated literals that do not occur complemented in *D*, as otherwise *D* cannot be of level *i*. Furthermore, one of the $K_i$ is the complement of *L*. But now, because  is false in  and  instead of deciding  the literal *L* should be propagated by a reasonable strategy. A contradiction. Note that none of the $K_i$ can be annotated with . $\square$

Many details are missing. To find the contradiction, we must show that there exists a state in the derivation with the trail $M_2K^\dag M_1$, and such that $D\vee L \in N \uplus U.$ The textbook does not explain why such a state is guaranteed to exist. Moreover, inductive reasoning is hidden under the ellipsis notation ($K_n\cdots K_2$). Such a high-level proof might be suitable for humans, but the details are needed in Isabelle, and Sledgehammer alone cannot fill in such large gaps, especially if induction is needed. The first version of the formal proof was over 700 lines long and is among the most difficult proofs we carried out.

We later refactored the proof. Following the book, each transition in  was normalized by applying  and  exhaustively. For example, we defined  so that  if  and  cannot be applied to  and  for some state *T*. However, normalization is not necessary. It is simpler to define  as , with the same condition on  as before. This change shortened the proof by about 200 lines. In a subsequent refactoring, we further departed from the book: We proved the invariant that all propagations have been performed before deciding a new literal. The core argument (“the literal *L* should be propagated by a reasonable strategy”) remains the same, but we do not have to reason about past transitions to argue about the existence of an earlier state. The invariant also makes it possible to generalize the statement of Theorem 10: We can start from any state that satisfies the invariant, not only from an initial state. The final version of the proof is 250 lines long.

Using Theorem 10 and assuming that only backjumping has a cost, we get a complexity of $\mathrm {O}(3^V)$, where *V* is the number of different propositional variables. If  is always preferred over  , the learned clause is never redundant in the sense of ordered resolution [57], yielding a complexity bound of $\mathrm {O}(2^V)$. We have not formalized this yet.

In  , and in our formalization, Theorem 10 is also used to establish the termination of  . However, the argument for the termination of  also applies to  irrespective of the strategy, a stronger result. To lift this result, we must show that  refines  .

### Connection with Abstract CDCL

It is interesting to show that  refines  , to establish beyond doubt that  is a CDCL calculus and to lift the termination proof and any other general results about  . The states are easy to connect: We interpret a  tuple  as a  pair , ignoring *C*.

The main difficulty is to relate the low-level conflict-related  rules to their high-level counterparts. Our solution is to introduce an intermediate calculus, called  , that combines consecutive low-level transitions into a single transition. This calculus refines both  and  and is sufficiently similar to  so that we can transfer termination and other properties from  to  through it.

Whenever the  calculus performs a low-level sequence of transitions of the form , the  calculus performs a single transition of a new rule that subsumes all four low-level rules:

  if

When simulating  in terms of  , two interesting scenarios arise. First,  ’s behavior may comprise a backjump: The rule can be simulated using  ’s  rule. The second scenario arises when the conflict clause is reduced to $\bot$, leading to a conclusive final state. Then,  has no counterpart in  . The two calculi are related as follows: If , either  or  is a conclusive state. Since  is well founded, so is  . This implies that  without  terminates.

Since  is mostly a rephrasing of  , it makes sense to restrict it to a *reasonable* strategy that prefers  and  over  , yielding  . The two strategy-restricted calculi have the same end-to-end behavior:


### A Strategy with Restart and Forget

We could use the same strategy for restarts as in Sect. 3.5, but we prefer to exploit Theorem 10, which asserts that no relearning is possible. Since only finitely many different duplicate-free clauses can ever be learned, it is sufficient to increase the number of learned clauses between two restarts to ensure termination. This criterion is the norm in modern SAT solvers. The lower bound on the number of learned clauses is given by an unbounded function . In addition, we allow an arbitrary subset of the learned clauses to be forgotten upon a restart but otherwise forbid  . The calculus  that realizes these ideas is defined by the two rules

  if  and
   if

**Fig. 2:**
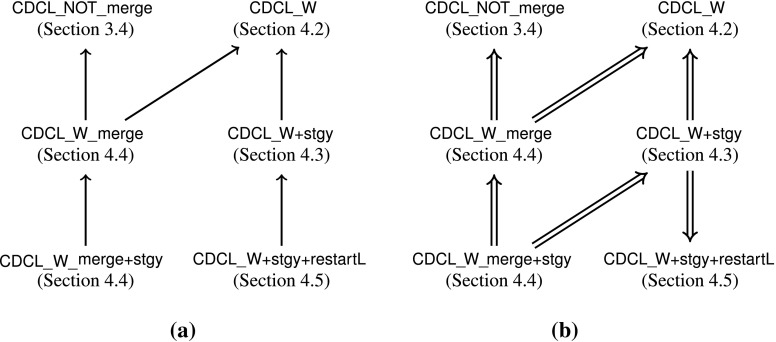
Connections involving the refined calculi. **a** Syntactic dependencies. **b** Refinements

We formally proved that  is totally correct. Figure 2 summarizes the situation, following the conventions of Fig. 1.

### Incremental Solving

SMT solvers combine a SAT solver with theory solvers (e.g., for uninterpreted functions and linear arithmetic). The main loop runs the SAT solver on a clause set. If the SAT solver answers “unsatisfiable,” the SMT solver is done; otherwise, the main loop asks the theory solvers to provide further, theory-motivated clauses to exclude the current candidate model and force the SAT solver to search for another one. This design crucially relies on incremental SAT solving: The possibility of adding new clauses to the clause set *C* of a conclusive satisfiable state and of continuing from there.

As a step towards formalizing SMT, we designed a calculus  that provides incremental solving on top of :

$$_{\,C}$$
  if $$M \not \vDash \lnot \, C$$ and
$$_{\,C}$$
  if $L M \vDash \lnot \, C$, $-L \in C$, $M'$ contains no literal of *C*, and

We first run the  calculus on a clause set *N*, as usual. If *N* is satisfiable, we can add a nonempty, duplicate-free clause *C* to the set of clauses and apply one of the two above rules. These rules adjust the state and relaunch .

#### Theorem 11

(Partial Correctness [20,  ]) If state  is conclusive and , then  is conclusive.

The key is to prove that the structural invariants that hold for  still hold after adding the new clause to the state. Then the proof is easy because we can reuse the invariants we have already proved about .

## A Naive Functional Implementation of CDCL

Sections 3 and 4 presented variants of DPLL and CDCL as parameterized transition systems, formalized using locales and inductive predicates. We now present a deterministic SAT solver that implements , expressed as a functional program in Isabelle.

When implementing a calculus, we must make many decisions regarding the data structures and the order of rule applications. Our functional SAT solver is very naive and does not feature any optimizations beyond those already present in the  calculus; in Sect. 6, we will refine the calculus further to capture the two-watched-literal optimization and present an imperative implementation relying on mutable data structures.

For our functional implementation, we choose to represent states by tuples , where propositional variables are coded as natural numbers and multisets as lists. Each transition rule in  is implemented by a corresponding function. For example, the function that implements the  rule is given below: 
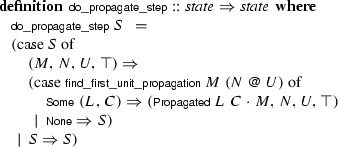


The functions corresponding to the different rules are combined into a single function that performs one step. The combinator  takes a list of functions implementing rules and tries to apply them in turn, until one of them has an effect on the state: 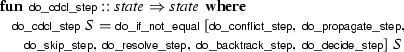 The main loop applies  until the transition has no effect: 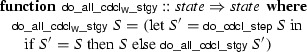 The main loop is a recursive program, specified using the  command [27]. For Isabelle to accept the recursive definition of the main loop as a terminating program, we must discharge a proof obligation stating that its call graph is well founded. This is a priori unprovable: The solver is not guaranteed to terminate if starting in an arbitrary state.

To work around this, we restrict the input by introducing a subset type that contains a strong enough structural invariant, including the duplicate-freedom of all the lists in the data structure. With the invariant in place, it is easy to show that the call graph is included in the  calculus, allowing us to reuse its termination argument. The partial correctness theorem can then be lifted, meaning that the SAT solver is a decision procedure for propositional logic.

The final step is to extract running code. Using Isabelle’s code generator [23], we can translate the program to Haskell, OCaml, Scala, or Standard ML. The resulting program is syntactically analogous to the source program in Isabelle, including its dependencies, and uses the target language’s facilities for datatypes and recursive functions with pattern matching. Invariants on subset types are ignored; when invoking the solver from outside Isabelle, the caller is responsible for ensuring that the input satisfies the invariant. The entire program is about 520 lines long in Standard ML. It is not efficient, due to its extensive reliance on lists, but it satisfies the need for a proof of concept.

## An Imperative Implementation of CDCL

As an impure functional language, Standard ML provides assignment and mutable arrays. We use these features to derive an imperative SAT solver that is much more efficient than the functional implementation. We start by integrating the two-watched-literal optimization into . Then we refine the calculus to apply rules deterministically, and we generate code that uses arrays to represent clauses and clause sets.

The resulting SAT solver is orders of magnitude faster than the naive functional implementation described in the previous section. However, it is one to two orders of magnitude slower than DPT 2.0 [21], the fastest imperative OCaml solver we know of, because it does not implement restarts or any sophisticated heuristics for learned clause minimization. We expect that many missing heuristics will be straightforward to implement. Due to inefficient memory handling, our solver is not competitive with state-of-the-art solvers.

### The Two-Watched-Literal Scheme

The two-watched-literal (2WL or TWL) scheme [42] is a data structure that makes it possible to efficiently identify candidate clauses for unit propagation and conflict. In each non-unit clause, we distinguish two *watched* literals—the other literals are *unwatched*. Initially, any of a non-unit clause’s literals can be chosen to be watched. In the simplest version of the scheme, the solver maintains the following invariant for each non-unit clause:

- ($\alpha$) A watched literal may be false only if all the unwatched literals are false.

As a consequence of this invariant, setting an unwatched literal will never yield a candidate for propagation or conflict, because the two watched literals can then only be true or unset.

For each literal *L*, the clauses that contain a watched *L* are chained together in a list (typically a linked list). When a literal *L* becomes true, the solver needs only to iterate through the list associated with $-L$ to find candidates for propagation or conflict. For each candidate clause, there are four possibilities:

1. If some of the unwatched literals are not false, we restore the invariant by *updating* the clause: We start watching one of the non-false unwatched literals instead of $-L$.
2. Otherwise, we consider the clause’s other watched literal:
  2.1. If it is not set, we can propagate it.
  2.2. If it is false, we have found a conflict.
  2.3. If it is true, there is nothing to do.

**Fig. 3:**
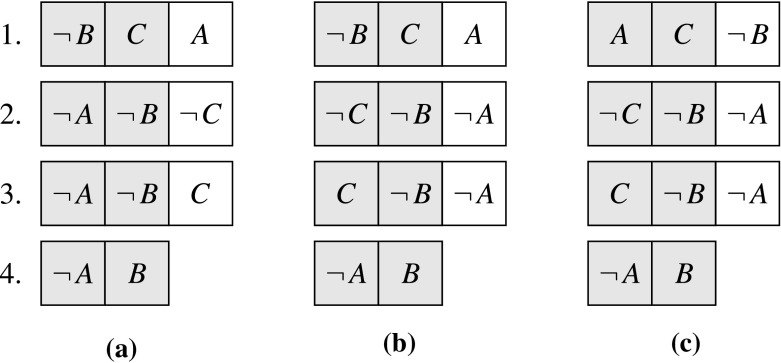
Evolution of the two-watched-literal data structure on an example

In  , a weaker invariant is used, inspired by MiniSat [18]:

- ($\beta$) A watched literal may be false only if the other watched literal is true or all the unwatched literals are false.

This invariant is easier to establish than ($\alpha$): If the other watched literal is true, there is nothing to do, regardless of the truth value of the unwatched literals. The four-step procedure above can easily be adapted, by pulling step 2.3 to the front.

To illustrate how the solver maintains the invariant, whether ($\alpha$) or ($\beta$), we consider the small problem shown in Fig. 3. The clauses are numbered from 1 to 4. Gray cells identify watched literals. Thus, clause 1 is $\lnot \,B\vee C \vee A$, where $\lnot \,B$ and *C* are watched.

1. We start with an empty trail and an arbitrary choice of watched literals (Fig. 3a).
2. We decide to make *A* true. The trail becomes $A^\dag$. In clauses 2 and 3, we exchange $\lnot \,A$ with another literal to restore the invariant (Fig. 3b).
3. We propagate *B* from clause 4. The trail becomes . In clause 1, we exchange $\lnot \,B$ with *A* to restore the invariant (Fig. 3c).
4. From clauses 2 and 3, we find out that we can propagate $\lnot \,C$ and *C*. We choose *C*. The trail becomes . Clause 2 is in conflict. The decision made in step 2 was wrong, so we backtrack.

Upon backtracking, there is no need to update the data structure. A key property for the data structure’s efficiency is that the invariant is preserved when we remove literals from the trail.

In MiniSat and other implementations, propagation is performed immediately whenever a suitable clause is discovered, and when a conflict is detected, the solver stops updating the data structure and processes the conflict. Using this more efficient strategy, the following scenario is possible for the example of Fig. 3:

1. We start with an empty trail and the same watched literals as before (Fig. 3a).
2. We decide to make *A* true. The trail becomes $A^\dag$.
3. We propagate *B* from clause 4. The trail becomes .
4. We propagate *C* from clause 3. The trail becomes . Clause 2 is in conflict. The decision made in step 2 was wrong, so we backtrack.

By making the right arbitrary choices, we could go from propagation to propagation without having to update the clauses. However, neither invariant holds for clauses 1 to 3 after step 3. To capture the new state of affairs, we need a more precise invariant and a richer notion of state that take into account any pending updates. The new invariant is as follows:

- ($\gamma$) If there are no pending updates for the clause and no conflict is being processed, invariant ($\beta$) holds.

An update is represented by a pair (*L*, *C*), where *L* is a literal that has become false and *C* is a clause that has *L* as one of its watched literals. Each time a literal *L* is added to the trail, all possible updates $(-L,\, C)$ are added to the set of pending updates, which is initially empty. Whenever a conflict is detected, the updates are reset to $\emptyset$. Pending updates can be processed at any time by the calculus.

### The CDCL Calculus with Watched Literals

CDCL with the 2WL data structure is defined as an abstract calculus  that refines . Nonunit clauses are represented as , where  is the multiset of watched literals (of cardinality 2) and  the multiset of unwatched literals. Unit clauses are represented as singleton multisets. The state must also keep track of pending updates. States have the form , where

- *M* is the trail;
- *N* is the initial nonunit clause set in 2WL format;
- *U* is the learned nonunit clause set in 2WL format;
- *D* is a conflict clause or $\top$;
is the initial unit clause set;
is the learned unit clause set;
is a multiset of literal–clause pairs (*L*, *C*) indicating that clause *C* must be updated with respect to literal *L*;
- *Q* is a set of literals for which further updates are pending.

and  do not influence the calculus; they are ghost components that are useful for connecting a 2WL state to the format expected by  :The  function converts a 2WL clause set to a standard clause set.

The first two rules of  have direct counterparts in  :

  if , $L'$ is not set in *M*, and
  if , $$-L' \in M$$, and

For both rules, the side condition  is necessary because invariant ($\beta$) is not required to hold for *C* while a (*L*, *C*) update is pending.

The next rules manipulate the state’s 2WL-specific components, without affecting the state’s semantics as seen through :

  if , and $N'$ and $U'$ are obtained from *N* and *U* by replacing  with
  if  and $$L' \in M$$

As in , propagations and conflicts are preferred over decisions. This is achieved by checking that  and $Q$ are empty when making a decision:

      if *L* is not defined in *M* and appears in *N*

The restriction on  is enough to ensure that the reasonable strategy is applied in  .  and  are as before, except that they also preserve the 2WL-specific components of the state. The  rule is replaced by two rules, because of the distinction between unit and nonunit clauses:

  if $D \ne \bot$ and *L* satisfies the conditions on
     if *L* satisfies the conditions on

#### Theorem 12

(Invariant [20, cdcl_twl_stgy_twl_struct_invs]) If state  satisfies invariant ($\gamma$) and , then *T* satisfies invariant ($\gamma$).

 refines  in the following sense:

#### Theorem 13

(Refinement [20, full_cdcl_twl_stgy_cdcl$_W$_stgy]) Let  be a state that satisfies invariant ($\gamma$). If , then 

 refines ’s end-to-end behavior and produces final states that are also final states for . We can apply Theorem 9 to establish partial correctness.

### Derivation of an Executable List-Based Program

The next step is to refine the calculus with watched literals to an executable program. The state is a tuple , where  is a list (instead of a set) of clauses containing first *n* initial nonunit clauses followed by the learned nonunit clauses, where clauses are represented as lists of literals starting with the watched ones; *M* uses indices in  to represent clause annotations; and  uses indices in  to represent clauses. The *D*, , , and *Q* components are as before.

The program’s main loop invokes functions that implement specific rules or set of rules. The function for  ,  ,  , and  is presented below: 
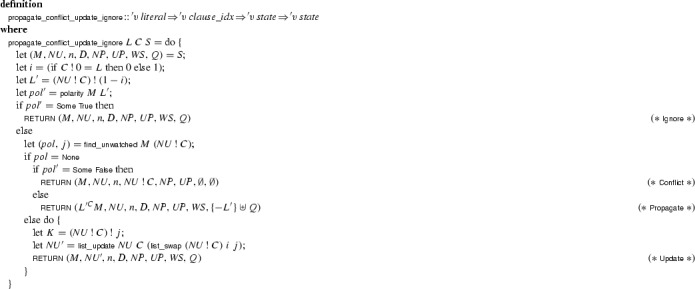
 The values  ,  , and  correspond to positive, negative, and undefined polarity, respectively. As we refine the program, we must provide additional invariants for the data structure—for example, indices in  are valid and  is a valid index. The assertion corresponding to the latter, , is not shown above, but it is needed for code generation.

The main loop is called . Although it imposes an order on rule applications, it is not fully deterministic—for example, it does not specify which literal to choose in  . The following theorem connects it to the  calculus:

#### Theorem 14

(Refinement [20,  ]) If  is a well-formed state and invariant ($\gamma$) holds for all clauses occurring in its  component, thenwhere  translates program states to  states.

The state returned by the program is final for  , which means by Theorem 13 that it is also final for  . We conclude that the program is a partially correct implementation of . In addition, since the specification always specifies a non-  result, the program always terminates normally.

In a further refinement step not presented here, we extend the state with watch lists that map from a literal to the clauses that are watched, instead of recalculating them each time. The watch lists are modeled by a function  such that  and update it in when required.

### Generation of Imperative Code

To be complete in a practical sense, an executable SAT solver must first initialize the 2WL data structure, run the  calculus, and return “satisfiable” (with a model) or “unsatisfiable,” depending on whether a conflict has been found. The initialization step is necessary not only to run the program on actual problems but also to ensure that it is possible to create a 2WL state that satisfies invariant ($\gamma$) for any input.

The input is a list of clauses, where each clause is itself a list. We require that the lists are nonempty and contain no duplicates. For each clause *C*, we perform the following steps:

1. If *C* is a unit clause *L*:
  1.1 Add *L* to the state’s  component.
  1.2 If $-L$ is in the trail, set the state’s *D* component to *L* and stop the procedure.
  1.3 Otherwise, add *L* to the state’s *M* and *Q* components, unless this has already been done.
2. Otherwise, add *C* to  Its first two literals are watched.

The result is a well-formed state that satisfies invariant ($\gamma$). If a conflict is found in step 1.2, the program can answer “unsatisfiable” immediately.

Before we can generate imperative code, we must first eliminate the remaining nondeterminism, notably the choice of literal in  . We implement the variable-move-to-front heuristic [5]. During initialization, we create a list containing all the literals. This list is used to initialize the doubly linked list needed by the heuristic. We also extract the maximal atom in the list to allocate the list of the polarity-checking optimization (Sect. 6.5) with the correct length.

Second, we must specify the data structures to use the generated code. Lists of clauses are refined to resizable arrays of nonresizable arrays. The dynamic aspect is required for adding learned clauses. Within a clause, only the order of literals needs to change. We had to formalize the data structure ourselves; for technical reasons, the resizable arrays from the Imperative Collection Framework [29, 31] cannot contain arrays. We were able to reuse some of the theorems proved on the separation logic level.

We used Sepref to refine the code of the SAT solver, including initialization. We restrict the type of the atoms  to natural numbers  . In our first version, we also used (unbounded) natural number to literals in the generated code: The literals  and  are encoded by the numbers $2\cdot i$ and $2\cdot i +1$, respectively. However, the extraction of an atom from the literals (the integer division by 2) was inefficient in Standard ML. Therefore, we changed our representation to 32-bits unsigned integers (so only $2^{31}$ atoms are allowed). The extraction of atoms now becomes bit-shifting.

The end-to-end refinement theorem, relating a semantic satisfiability check on the input problem ( that returns  if unsatisfiable) to the Imperative HOL heap code (), is stated below, where the  relation refines a multiset of multisets of literals to a list of lists of 32-bit unsigned integers, and the  relation refines the model that is returned as a list of literals.

#### Theorem 15

(End-to-End Correctness [20,  ])

The following refinement relation holds: 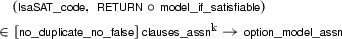


### Fast Polarity Checking

The imperative code described in the previous subsection suffers from a crippling inefficiency: The solver often needs to compute the polarity of a literal, and currently this is achieved by traversing the trail *M*, which may be very large. In practice, solvers employ a map from atoms to their current polarity.

Using stepwise refinement, we integrate this optimization into the imperative data structure used for the trail. This refinement step is isolated from the rest of the development, which only relies on its final result: a more efficient implementation of the trail and its operations. As Lammich observed elsewhere [32], this kind of modularity is invaluable when designing complex data structures.

Since the atoms are natural numbers, we enrich the trail data structure with a list of polarities (of type ), such that the $(i + 1)$st element gives the polarity of atom *i*. The new  function is defined as follows: 
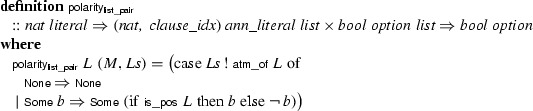


Given $N_1$ the set of all valid literals (i.e., the positive and negative version of all atoms that appear in the problem), the refinement relation between the trail with the list of polarities and the simple trail is defined as follows: 
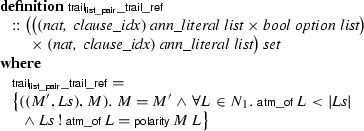
 This invariant ensures that the list  is long enough and contains the polarities. We can link the new polarity function to the simpler one. If , then

**Figure:**
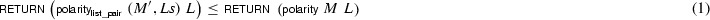

In a subsequent refinement step, we use Sepref to implement the list of polarities by an array, and atoms are mapped to 32-bits unsigned integers (  ), as in Sect. 6.4. Accordingly, we define two auxiliary relations:

- The relation  refines a literal with natural number atoms by a literal encoded as a 32-bit unsigned integer.
- The relation  refines the trail data structure to use an array of polarities (instead of a list) and annotated literals of type  , using the 32-bit representation of literals. The clause indices of type  remain unbounded unsigned integers.

Sepref generates the imperative program  and derives the following refinement theorem:

**Figure:**
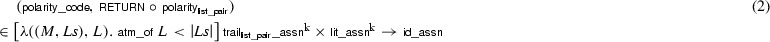

The precondition, in square brackets, ensures that we can only take the polarity of a literal that is within bounds. The term after the arrow is the refinement for the result, which is trivial here because the data structure for polarities remains .

Composing the refinement steps (1) and (2) yields the theorem 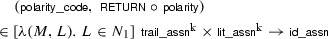
where  combines the two refinement relations for trails  and . The precondition  is a consequence of $L \in N_1$ and the invariant . If we invoke Sepref now and discharge  ’s preconditions, all occurrences of the unoptimized  function are be replaced by  . After adapting the initialization to allocate the array for  of the correct size, we can prove end-to-end correctness as before with respect to the optimized code (cf. Theorem 15).

## Discussion and Related Work

Our formalization of the DPLL and CDCL calculi consists of about 28 000 lines of Isabelle text. The work was done over a period of 10 months almost entirely by Fleury, who also taught himself Isabelle during that time. It covers nearly all of the metatheoretical material of Sections 2.6 to 2.11 of  and Section 2 of Nieuwenhuis et al., including normal form transformations and ground unordered resolution [19]. The refinement to an imperative program is about 20 000 lines long and took about 6 months to perform.

It is difficult to quantify the cost of formalization as opposed to paper proofs. For a sketchy argument, formalization may take an arbitrarily long time; indeed, Weidenbach’s eight-line proof of Theorem 10 initially took 700 lines of Isabelle. In contrast, given a very detailed paper proof, one can sometimes obtain a formalization in less time than it took to write the paper proof [60]. A frequent hurdle to formalization is the lack of suitable libraries. We spent considerable time adding definitions, lemmas, and automation hints to Isabelle’s multiset library, and the refinement to resizable arrays of arrays required an elaborate setup, but otherwise we did not need any special libraries. We also found that organizing the proof at a high level, especially locale engineering, is more challenging, and perhaps even more time consuming, than discharging proof obligations.

One of our initial motivations for using locales, besides the ease with which it lets us express relationships between calculi, was that it allows abstracting over the concrete representation of the state. However, we discovered that this is often too restrictive, because some data structures need sophisticated invariants, which we must establish at the abstract level. We found ourselves having to modify the base locale each time we attempted to refine the data structure, an extremely tedious endeavor.

In contrast, the Refinement Framework, with its focus on functions, allows us to exploit local assumptions. Consider the  function (Sect. 3.2), which adds a literal to the trail. Whenever the function is called, the literal is not already set and appears in the clauses. The polarity-checking optimization (Sect. 6.5) relies on the latter property to avoid checking bounds when updating the atom-to-polarity map. With the Refinement Framework, there are enough assumptions in the context to establish the property. With a locale, we would have to restrict the specification of  to handle only those cases where the literals is in the set of clauses, leading to changes in the locale definition itself and to all its uses, well beyond the polarity-checking code.

While refining to the heap monad, we discovered several issues with our program. We had forgotten several assertions (especially array bound checks) and sometimes mixed up the  and  annotations, resulting in large, hard-to-interpret proof obligations. Sepref is a very useful tool, but it provides few safeguards or hints when something goes wrong. Moreover, the Isabelle/jEdit user interface can be unbearably slow at displaying large proof obligations.

Given the varied level of formality of the proofs in the draft of  , it is unlikely that Fleury will ever catch up with Weidenbach. But the insights arising from formalization have already enriched the textbook in many ways. For the calculi described in this paper, the main issues were that fundamental invariants were omitted and some proofs may have been too sketchy to be accessible to the book’s intended audience. We also found a major mistake in an extension of CDCL using the branch-and-bound principle: Given a weight function, the calculus aims at finding a model of minimal weight. In the course of formalization, Fleury came up with a counterexample that invalidates the main correctness theorem, whose proof confused partial and total models.

For discharging proof obligations, we relied heavily on Sledgehammer, including its facility for generating detailed Isar proofs [10] and the SMT-based *smt* tactic [13]. We found the SMT solver CVC4 particularly useful, corroborating earlier empirical evaluations [50]. In contrast, the counterexample generators Nitpick and Quickcheck [8] were seldom useful. We often discovered flawed conjectures by observing Sledgehammer fail to solve an easy-looking problem. As one example among many, we lost perhaps one hour working from the hypothesis that converting a set to a multiset and back is the identity. Because Isabelle’s multisets are finite, the property does not hold for infinite sets *A*; yet Nitpick and Quickcheck fail to find a counterexample, because they try only finite values for *A* (and Quickcheck cannot cope with underspecification anyway).

At the calculus level, we followed Nieuwenhuis et al. (Sect. 3) and Weidenbach (Sect. 4), but other accounts exist. In particular, Krstić and Goel [28] present a calculus that lies between  and  on a scale from abstract to concrete. Unlike Nieuwenhuis et al., they have a concrete  rule. On the other hand, whereas Weidenbach only allows to resolve the conflict (  ) with the clause that was used to propagate a literal, Krstić and Goel allow any clause that could have cause the propagation (rule  ). Another difference is that their  and  rules must explicitly check that no clause is learned twice (cf. Theorem 10).

Formalizing metatheoretical results about logic in a proof assistant is an enticing, if somewhat self-referential, prospect. Shankar’s proof of Gödel’s first incompleteness theorem [52], Harrison’s formalization of basic first-order model theory [24], and Margetson and Ridge’s formalized completeness and cut elimination theorems [36] are some of the landmark results in this area. Recently, SAT solvers have been formalized in proof assistants. Marić [37, 38] verified a CDCL-based SAT solver in Isabelle/HOL, including two watched literals, as a purely functional program. The solver is monolithic, which complicates extensions. In addition, he formalized the abstract CDCL calculus by Nieuwenhuis et al. and, together with Janičić [37, 39], the more concrete calculus by Krstić and Goel [28]. Marić’s methodology is quite different from ours, without the use of refinements, inductive predicates, locales, or even Sledgehammer.

In his Ph.D. thesis, Lescuyer [34] presents the formalization of the CDCL calculus and the core of an SMT solver in Coq. He also developed a reflexive DPLL-based SAT solver for Coq, which can be used as a tactic in the proof assistant. Another formalization of a CDCL-based SAT solver, including termination but excluding two watched literals, is by Shankar and Vaucher in PVS [53]. Most of this work was done by Vaucher during a two-month internship, an impressive achievement. Finally, Oe et al. [47] verified an imperative and fairly efficient CDCL-based SAT solver, expressed using the Guru language for verified programming. Optimized data structures are used, including for two watched literals and conflict analysis. However, termination is not guaranteed, and model soundness is achieved through a run-time check and not proved.

## Conclusion

The advantages of computer-checked metatheory are well known from programming language research, where papers are often accompanied by formalizations and proof assistants are used in the classroom [44, 49]. This article, like its predecessors and relatives [9, 12, 51], reported on some steps we have taken to apply these methods to automated reasoning. Compared with other application areas of proof assistants, the proof obligations are manageable, and little background theory is required.

We presented a formal framework for DPLL and CDCL in Isabelle/HOL, covering the ground between an abstract calculus and a verified imperative SAT solver. Our framework paves the way for further formalization of metatheoretical results. We intend to keep following  , including its generalization of ordered ground resolution with CDCL, culminating with a formalization of the full superposition calculus and extensions. Thereby, we aim at demonstrating that interactive theorem proving is mature enough to be of use to practitioners in automated reasoning, and we hope to help them by developing the necessary libraries and methodology.

The CDCL algorithm, and its implementation in highly efficient SAT solvers, is one of the jewels of computer science. To quote Knuth [26, p. iv], “The story of satisfiability is the tale of a triumph of software engineering blended with rich doses of beautiful mathematics.” What fascinates us about CDCL is not only how or how well it works, but also why it works so well. Knuth’s remark is accurate, but it is not the whole story.
